# The triglyceride-glucose index in relation to psychotic symptoms in adolescents with major depressive disorder

**DOI:** 10.3389/fpsyt.2026.1755283

**Published:** 2026-01-28

**Authors:** Yi Tang, Wen Wu, Yun Zhang, Ji Yin, Dan Luo, Zhangyan Zhou, Hansong Xu

**Affiliations:** 1 Department of Clinical Nutrition, The Second People’s Hospital of Guizhou Province, Guiyang, China; 2 Health Management Center, The Affiliated Hospital of Guizhou Medical University, Guiyang, China; 3 Department of Endocrinology, The Second People’s Hospital of Guizhou Province, Guiyang, China; 4 Department of Psychiatry of Women and Children, The Second People’s Hospital of Guizhou Province, Guiyang, China

**Keywords:** adolescents, insulin resistance, major depressive disorder, psychotic symptoms, triglyceride glucose index

## Abstract

**Objective:**

It is increasingly recognized that insulin resistance (IR) is associated with major depressive disorder(MDD) and a spectrum of psychotic symptoms. However, a paucity of data exists regarding the connection of IR with concurrent psychotic symptoms in adolescent MDD patients. Consequently, we aimed to examine the correlation of the triglyceride-glucose index (TyG), an alternative measure of IR, with psychotic symptoms among adolescents presenting with MDD.

**Methods:**

The study included 1,556 adolescents aged 13–17 years with depressive disorders. Demographic data were collected, and psychotic symptoms were assessed through clinical interviews. Assessment of depressive and anxiety symptoms was conducted with the 17-item Hamilton Depression Rating Scale (HAMD-17) and the 14-item Hamilton Anxiety Rating Scale (HAMA). Levels of fasting blood glucose (FBG), triglycerides (TG), and other serum markers were determined. The relationship between the TyG index and psychotic symptoms was subsequently investigated by applying multivariable binary logistic regression, restricted cubic spline (RCS), and threshold effect analyses.

**Results:**

A total of 1,556 patients were included in this study, with 1,158 females (74.4%) and 398 males (25.6%). Among all participants, 402 (25.8%) exhibited psychotic symptoms. A positive association persisted between the TyG index and psychotic symptoms after comprehensive covariate adjustment, demonstrating a 98.2% increase in odds per 1-unit increment in the index (OR = 1.982; 95% CI: 1.499, 2.620). Compared to the reference tertile (T1), participants in the highest TyG tertile (T3) exhibited significantly greater odds of psychotic symptoms (OR = 2.138; 95% CI: 1.526, 2.994). Furthermore, multivariable RCS analysis established that this relationship was nonlinear in nature (p = 0.045). Subsequent analysis pinpointed a TyG index of 8.06 as a critical threshold, beyond which the risk of psychotic symptoms emerged as significant (OR = 1.618, 95% CI: 1.108-2.363).

**Conclusions:**

Analysis of the dose-response relationship revealed a J-shaped curve linking the TyG index to psychotic symptoms among adolescents with MDD, characterized by a threshold value near 8.06.

## 1 Introduction

Major depressive disorder (MDD), particularly during severe episodes characterized by psychotic features, is marked not only by core symptoms such as profound mood disturbance, diminished interest, and reduced energy but may also manifest with psychotic symptoms including hallucinations and/or delusions (1). Previous studies have reported that the prevalence of psychotic symptoms in patients with MDD ranges from 5.3% to 52.7% (2–4). The prevalence is higher in hospitalized patients compared to outpatients, and generally higher in adolescents than in adults (5). Existing studies have confirmed that patients with MDD and psychotic symptoms have a higher suicide risk and poorer social functioning outcomes (6, 7). Compared to adults, adolescence, as a critical period of physical and psychological development, is characterized by a brain that is particularly sensitive to metabolic and endocrine changes (8). Depression can be viewed as a metabolic stress response affecting multiple bodily systems, and adolescents with psychotic symptoms typically experience a poorer clinical prognosis. Therefore, it is necessary to comprehensively investigate the key factors associated with psychotic symptoms in this population.

In recent years, the role of metabolic abnormalities in the pathophysiology of depression has garnered increasing attention, with insulin resistance (IR) being considered a key link between mental disorders and physical health (9–13). IR can drive the pathophysiological process of depression by altering the regulatory function of the hypothalamic-pituitary-adrenal (HPA) axis, leading to elevated cortisol levels (11). It is noteworthy that the presence of IR may be associated with more severe depressive symptoms and cognitive dysfunction, and it serves as an important predictor of treatment resistance in patients undergoing first-line antidepressant therapies, such as selective serotonin and norepinephrine reuptake inhibitors (9). However, in clinical practice, the gold standard methods for assessing IR are complex and not easily applicable. The triglyceride-glucose (TyG) index, a readily available composite of fasting triglycerides and glucose, serves as a robust surrogate for IR. Growing evidence underscores its substantial value in research on adult psychotic depression (14, 15). This suggests that metabolic dysregulation, as represented by the TyG index, may be a key biological pathway driving the development of MDD, particularly its psychotic subtype.

Despite evidence suggesting that metabolic dysregulation, particularly IR, plays a significant role in the pathophysiology of psychotic depression, and that the TyG index is an effective tool for evaluating this condition, most relevant studies have focused on adult populations (16, 17). Given the unique development of the brain, metabolism, and endocrine system during adolescence, the underlying pathophysiological mechanisms may differ from those in adults (8, 18). Therefore, directly applying findings from adult studies to adolescent populations may not be appropriate. A critical gap exists in the current literature regarding the association of the TyG index with psychotic symptoms specifically in the adolescent MDD population. Based on this, we hypothesize that adolescents with MDD and psychotic symptoms will have significantly higher TyG indices compared to those without such symptoms. This study aims to test this hypothesis and to explore the association between the TyG index and psychotic symptoms in adolescents with MDD.

## 2 Methods

### 2.1 Participants

This cross-sectional study was carried out at The Second People’s Hospital of Guizhou Province in Guiyang, China, from August 2023 to August 2025. The institutional ethics committee granted approval (IRB: 2023-79), and all procedures complied with the tenets of the Declaration of Helsinki. Eligibility criteria: (1) age 13–17 years; and (2) a diagnosis of MDD based on the International Classification of Diseases, 10th edition (ICD-10); (3) Currently not using psychiatric medications (e.g., aripiprazole, risperidone, etc.); (4) Have received at least 6 years of formal education; (5) Each participant was enrolled in the study only after obtaining informed consent from their legal guardian. Exclusion criteria comprised: (1) major physical comorbidities (e.g., HIV/AIDS, heart disease); (2) diagnosis of a serious neurological disorder, for instance, epilepsy or traumatic brain injury; (3) a documented history of severe psychiatric illness, including bipolar disorder or schizophrenia. Upon obtaining informed consent, all participants were assessed by trained professionals.

### 2.2 Demographic characteristics

The demographic data in this study were obtained by trained research staff and encompassed gender, age, as well as smoking and drinking history (smoking history: any history of tobacco use prior to enrollment; drinking history: any history of alcohol consumption prior to enrollment).

### 2.3 Clinical measurements

Assessment of blood pressure was conducted after participants had rested seated for at least 15 minutes. Two separate readings were obtained from the right arm with a standard mercury sphygmomanometer, and the final systolic blood pressure (SBP) and diastolic blood pressure (DBP) values were subsequently derived from the average of these two measurements. Assessment of height and weight was conducted using standardized methods. The Body Mass Index (BMI) was determined using the standard equation: weight (kg)/[height (m)]².

The psychotic symptoms of participants were assessed through clinical interviews conducted by experienced psychiatrists. The diagnosis was based on evaluations of delusions (excluding exaggerated delusions) and hallucinations. To ensure comprehensive evidence, when participants were unable to provide clear information, accompanying individuals were interviewed when necessary to gather additional details. The severity of symptoms was scored according to the following criteria: 0 = not present, 1 = suspected, 2 = present, 3 = clearly present, 4 = certainly present and dominating behavior. Ultimately, a total score of ≤1 was considered indicative of the absence of psychotic symptoms, while a score of ≥2 indicated the presence of psychotic symptoms. Thus, the primary outcome for analysis was a binary variable (psychotic symptoms: present vs. absent).

Depressive and anxiety symptoms were evaluated using the 17-item Hamilton Depression Rating Scale (HAMD-17) (19) and the 14-item Hamilton Anxiety Rating Scale (HAMA) (20), respectively. Scores on the HAMD-17 range from 0 to 52, serving as a direct indicator of depressive symptom severity. For the HAMA, the total score spans 0 to 56, and an increased score is indicative of greater anxiety severity.

### 2.4 Blood samples

After a minimum 8-hour overnight fast, venous blood was drawn in the morning for biochemical profiling. A panel of laboratory parameters, including Red blood cells (RBC), White blood cells (WBC), Hemoglobin (Hb), Serum uric acid (SUA), Fasting blood glucose (FBG), Total cholesterol (TC), Triglycerides (TG), High-density lipoprotein cholesterol (HDL-C), and Low-density lipoprotein cholesterol (LDL-C), was assayed by the central laboratory. From these, the TyG index was derived from the equation: ln [fasting TG (mg/dL) × FBG (mg/dL)/2] (21).

### 2.5 Statistical analysis

Categorical variables are summarized by counts and percentages (%), with group differences examined by Chi-square tests. Description of continuous data utilized mean (SD) for normally distributed variables and median (IQR) for skewed variables. Comparison across groups was performed with either one-way ANOVA or the Kruskal-Wallis H test, selected based on data distribution.

We employed logistic regression to evaluate the associations of the continuous and tertile-based TyG index with psychotic symptoms, expressing results as ORs with 95% CIs. To address confounding, we designed sequential models: Model 1 incorporated age and sex; Model 2 cumulatively included smoking, alcohol consumption, SBP, and DBP; Model 3 included comprehensive adjustments, incorporating variables such as BMI, HAMD-17, HAMA, blood cell counts (red and white), Hb, SUA, TC, HDL-C, and LDL-C. The covariates for Model 3 were selected *a priori* based on established literature regarding potential confounders in the relationship between metabolic markers and psychiatric outcomes (16, 22). Specifically, demographics (age, sex) and core metabolic parameters (e.g., BMI, lipids) were considered fundamental confounders. Clinical scores HAMD–17, HAMA were included to adjust for overall non-psychotic symptoms severity, thereby aiming to isolate the association of TyG with psychotic symptoms specifically.

Restricted cubic spline (RCS) models with four knots were fitted to elucidate the dose-response relationship between the TyG index and psychotic symptoms, incorporating adjustments from Model 3. Subsequent piecewise logistic regression aimed to identify critical thresholds where the association magnitude changed significantly, providing data on risk-associated TyG levels for potential clinical application.

Statistical analyses were performed using R (Version 4.5.0; The R Foundation; http://www.R-project.org) and Free Statistics software (Version 1.9). Statistical significance was defined as a *p* value of less than 0.05 based on two-sided tests.

## 3 Results

### 3.1 Baseline characteristics

We enrolled a total of 1,556 patients in this study. Among these participants, the median age was 16.0 years (IQR 15.0-17.0), and the gender composition was 25.6% (n=398) male and 74.4% (n=1158) female. A prevalence of 25.8% for psychosis symptoms was found among the study participants. Comparative analysis of participant demographics across TyG index tertiles is summarized in **Table 1**. Discernible variations were noted among the tertile groups for psychotic symptoms, SBP, DBP, BMI, HAMD-17, HAMA, RBC, WBC, HB, SUA, FBG, TC, TG, HDL-c, and LDL-c (all *p* < 0.05). In contrast, baseline profiles for gender, age, smoking status, and alcohol use remained comparable across all TyG strata (all *p* > 0.05).

**Table 1:** Baseline characteristics of participants stratified by TyG index.

Variables	Total (n=1556)	TyG index			*p*-value
		T1 (6.88-8.10) (n=519)	T2 (8.11-8.53) (n=519)	T3 (8.54-10.92) (n=518)	
Gender					0.254
Male	398 (25.6)	121 (23.3)	133 (25.6)	144 (27.8)	
Female	1158 (74.4)	398 (76.7)	386 (74.4)	374 (72.2)	
Age(years)	16.0 (15.0,17.0)	16.0 (15.0,17.0)	16.0 (15.0,17.0)	16.0 (14.0,17.0)	0.136
Smoking status					0.066
without	1487 (95.6)	504 (97.1)	495 (95.4)	488 (94.2)	
with	69 (4.4)	15 (2.9)	24 (4.6)	30 (5.8)	
Alcohol status					0.576
without	1504 (96.7)	505 (97.3)	501 (96.5)	498 (96.1)	
with	52 (3.3)	14 (2.7)	18 (3.5)	20 (3.9)	
Systolic blood pressure (mmHg)	113.34 ± 11.60	111.78 ± 11.38	113.06 ± 12.21	115.19 ± 10.93	<0.001
Diastolic blood pressure (mmHg)	73.27 ± 8.67	72.50 ± 8.97	73.47 ± 8.24	73.83 ± 8.76	0.038
Body mass index (kg/m^2^)	20.69 ± 3.82	19.48 ± 2.70	20.47 ± 3.33	22.12 ± 4.68	<0.001
Psychotic symptoms					<0.001
Without	1154 (74.2)	429 (82.7)	381 (73.4)	344 (66.4)	
With	402 (25.8)	90 (17.3)	138 (26.6)	174 (33.6)	
Hamilton Depression Rating Scale-17	26.26 ± 3.39	25.38 ± 3.37	26.14 ± 3.06	27.26 ± 3.45	<0.001
Hamilton Anxiety Rating Scale	22.97 ± 4.71	22.20 ± 4.74	22.95 ± 4.69	23.76 ± 4.57	<0.001
Red blood cells (×10^12^/L)	4.60 ± 0.56	4.52 ± 0.56	4.61 ± 0.57	4.68 ± 0.55	<0.001
White blood cells (×10^9^/L)	5.95 ± 1.52	5.68 ± 1.51	5.86 ± 1.44	6.30 ± 1.54	<0.001
Hemoglobin (g/L)	133.88 ± 16.69	131.28 ± 17.06	134.46 ± 15.47	135.91 ± 17.17	<0.001
Serum uric acid (μmol/L)	349.90 ± 92.70	331.25 ± 82.75	343.57 ± 88.63	374.91 ± 88.51	<0.001
Fasting blood glucose (mmol/L)	4.96 ± 0.53	4.79 ± 0.41	4.96 ± 0.42	5.13 ± 0.66	<0.001
Total cholesterol (mmol/L)	3.88 ± 0.78	3.62 ± 0.76	3.85 ± 0.71	4.16 ± 0.77	<0.001
Triglycerides (mmol/L)	1.21 ± 0.73	0.67 ± 0.14	1.05 ± 0.15	1.92 ± 0.84	<0.001
High-density lipoprotein cholesterol (mmol/L)	1.15 ± 0.32	1.24 ± 0.31	1.17 ± 0.33	1.05 ± 0.29	<0.001
Low-density lipoprotein cholesterol (mmol/L)	2.36 ± 0.74	2.12 ± 0.64	2.37 ± 0.79	2.58 ± 0.72	<0.001

The variables are presented as n (%) or the mean ± SD or median (quartile 1-quartile 3).

### 3.2 Association of covariates and psychotic symptoms

**Table 2** presents the results of the univariate logistic regression analysis exploring the associations between covariates and psychotic symptoms. Significant associations were identified between psychotic symptoms and several variables, including BMI (OR = 1.034, 95% CI: 1.005, 1.064), HAMD-17 (OR = 1.034, 95% CI: 1.001, 1.069), HAMA (OR = 1.037, 95% CI: 1.013, 1.063), FBG (OR = 1.417, 95% CI: 1.132, 1.773), TC (OR = 1.202, 95% CI: 1.041, 1.388), and TG (OR = 1.534, 95% CI: 1.319, 1.786). A significant inverse relationship was identified for age, where each unit increase was associated with reduced odds of psychotic symptoms (OR = 0.895, 95% CI: 0.820, 0.976; *p* < 0.05).

**Table 2:** Association of covariates and psychotic symptoms.

Covariates	OR(95%CI)	*p*-value
Gender		
Male	1 (Ref)	
Female	1.151 (0.883,1.500)	0.299
Age (years)	0.895 (0.820,0.976)	0.012
Smoking status		
without	1 (Ref)	
with	0.592 (0.314,1.115)	0.105
Alcohol status		
without	1 (Ref)	
with	0.675 (0.336,1.359)	0.271
Systolic blood pressure (mmHg)	1.005 (0.995,1.015)	0.323
Diastolic blood pressure (mmHg)	1.002 (0.989,1.015)	0.743
Body mass index (kg/m^2^)	1.034 (1.005,1.064)	0.023
Hamilton Depression Rating Scale-17	1.034 (1.001,1.069)	0.043
Hamilton Anxiety Rating Scale	1.037 (1.013,1.063)	0.003
Red blood cells (×10^12^/L)	1.022 (0.835,1.250)	0.835
White blood cells (×10^9^/L)	1.069 (0.994,1.149)	0.073
Hemoglobin (g/L)	0.996 (0.990,1.003)	0.275
Serum uric acid (μmol/L)	1.000 (0.999,1.002)	0.456
Fasting blood glucose (mmol/L)	1.417 (1.132,1.773)	0.002
Total cholesterol (mmol/L)	1.202 (1.041,1.338)	0.012
Triglycerides (mmol/L)	1.534 (1.319,1.786)	<0.001
High-density lipoprotein cholesterol (mmol/L)	0.788 (0.548,1.134)	0.199
Low-density lipoprotein cholesterol (mmol/L)	1.116 (0.963,1.295)	0.145

OR, odds ratio; CI, confidence interval; Ref, reference.

### 3.3 Relationship between TyG index and psychotic symptoms

Analysis of TyG index tertiles (Tertile 1 [T1], Tertile 2 [T2], and Tertile 3 [T3]) within the fully adjusted Model 3 revealed a gradient of risk for psychotic symptoms. Relative to the T1 reference group, significantly greater odds were observed for T2 (OR = 1.690, 95% CI: 1.242, 2.299) and T3 (OR = 2.138, 95% CI: 1.526, 2.994), indicating increased likelihood with progressively higher TyG index tertiles (**Table 3**). This aligns with the significant positive association found for the continuous TyG index in the same model (OR = 1.982, 95% CI: 1.499, 2.620).

**Table 3:** Analysis between TyG index and psychotic symptoms.

Variables	Crude OR (95% CI)	*P*-value	Model 1 OR (95% CI)	*P*-value	Model 2 OR (95% CI)	*P*-value	Model 3 OR (95% CI)	*P*-value
TyG index	2.078 (1.657, 2.606)	<0.001	2.075 (1.653, 2.606)	<0.001	2.073 (1.648, 2.606)	<0.001	1.982 (1.499, 2.620)	<0.001
Categories								
T1	1 (Ref)		1 (Ref)		1 (Ref)		1 (Ref)	
T2	1.727 (1.280, 2.329)	<0.001	1.726 (1.279, 2.329)	<0.001	1.732 (.282, 2.339)	<0.001	1.690 (1.242, 2.299)	0.001
T3	2.411 (1.802, 3.227)	<0.001	2.393 (1.786, 3.206)	<0.001	2.398 (1.786, 3.220)	<0.001	2.138 (1.526, 2.994)	<0.001
Trend test	1.540 (1.335, 1.777)	<0.001	1.535 (1.329, 1.771)	<0.001	1.536 (1.329, 1.775)	<0.001	1.454 (1.231, 1.717)	<0.001

OR, odds ratio; CI, confidence interval; Ref, reference.

Model 1: Adjusted for age, gender.

Model 2: Adjusted as for model 1, additionally adjusted for smoking, alcohol, systolic blood pressure, and diastolic blood pressure.

Model 3: Adjusted as for model 2, additionally adjusted for body mass index, Hamilton Depression Rating Scale-17, Hamilton Anxiety Rating Scale, red blood cells, white blood cells, hemoglobin, Serum uric acid, total cholesterol, high-density lipoprotein cholesterol, low-density lipoprotein cholesterol.

### 3.4 The non-linear association between TyG index and psychotic symptoms

**Figure 1:**
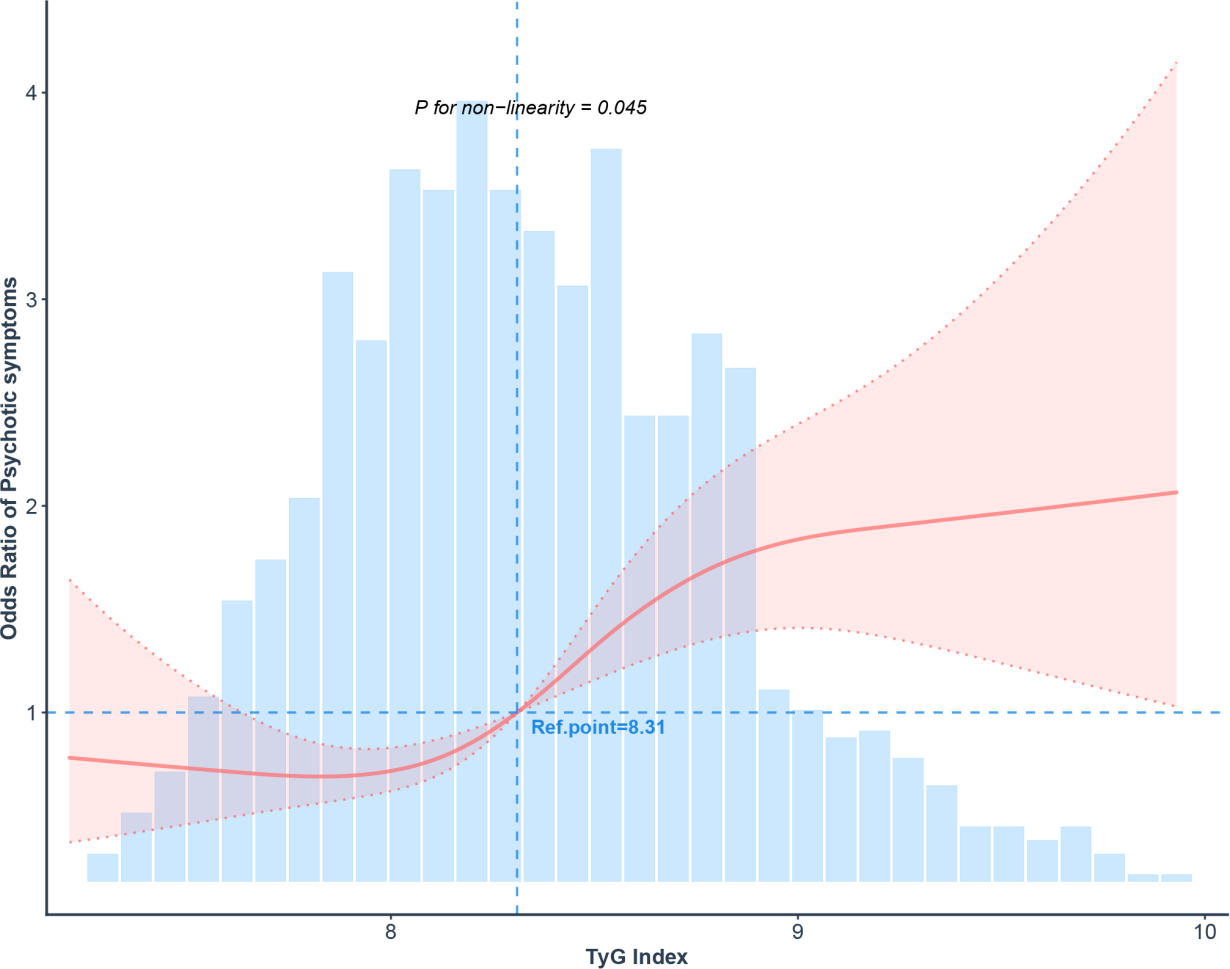
The non-linear association between the TyG index and psychotic symptoms is depicted, with the solid line representing the predicted values and the dashed lines indicating the 95% confidence intervals. These estimates are adjusted based on model 3 in **Table 3**.

RCS analysis was conducted to investigate the dose-response pattern. After accounting for confounders in Model 3, the relationship between the TyG index and psychotic symptoms demonstrated a significant departure from linearity (P-non-linear = 0.045; **Figure 1**). Threshold analysis revealed an inflection point at a TyG index value of 8.06. Our findings indicated that for TyG index values above this threshold, the risk of psychotic symptoms increased with higher TyG index values (OR = 1.622, 95% CI: 1.110, 2.369). More precisely, a one-unit rise in the TyG index was associated with a 62.2% increase in the likelihood of psychotic symptoms. Below this threshold (TyG index < 8.06), the relationship with psychotic symptoms was inverse (**Figure 1**), and subsequent threshold analysis confirmed no significant link between the index and symptom risk within this range (**Table 4**).

**Table 4:** Threshold effect analysis of TyG index and psychotic symptoms.

TyG index	OR (95%CI)	*P*-value
<8.06	0.331 (0.103-1.060)	0.0627
≥8.06	1.622 (1.110-2.369)	0.0124
Likelihood ratio		<0.001

Adjusted for age, gender, smoking, alcohol, systolic blood pressure, diastolic blood pressure, body mass index, Hamilton Depression Rating Scale-17, Hamilton Anxiety Rating Scale, red blood cells, white blood cells, hemoglobin, Serum uric acid, total cholesterol, high-density lipoprotein cholesterol, low-density lipoprotein cholesterol. Only 95% of the data is displayed.

TyG index, triglyceride-glucose index; OR, odds ratio; CI, confidence interval.

## 4 Discussion

Our findings reveal a J-shaped curve characterizing the TyG index-psychotic symptom relationship in adolescent MDD, pivoting at a TyG value of 8.06. A significant positive association was present only above this point, with a 61.8% increased risk per unit. This non-linear dynamic suggests the TyG index’s utility in pinpointing MDD adolescents at heightened psychosis risk, thereby aiding targeted clinical strategies. The biological foundations of this association demand further investigation.

This identified inflection point (TyG=8.06) provides a preliminary clinical reference threshold, corroborating existing evidence of a critical TyG range associated with heightened psychotic risk (16). Monitoring the TyG index in clinical practice, especially when values approach this threshold, could aid in early identification of at-risk adolescents with MDD for targeted metabolic and psychiatric interventions.

This study found that the comorbidity rate of psychotic symptoms in adolescents with MDD is 25.8%, which falls within the range reported in the existing literature, with prevalence rates varying from 9.8% to 52.7% across different studies (3–5, 23, 24). The differences in reported prevalence rates may be attributed to methodological variations and population heterogeneity across studies. First, discrepancies in diagnostic tools and criteria can directly impact the identification of psychotic symptoms. Second, there are substantial differences in the study populations: some studies included only first-episode, untreated hospitalized patients, while others mixed hospitalized, outpatient, and community populations, the latter of which may have a longer disease duration and a more complex medication history (16). Furthermore, factors such as age, gender, geography, and sample size may contribute to the observed inconsistencies in the results. Nevertheless, these findings collectively underscore a key clinical fact: the co-occurrence of psychotic symptoms in patients with MDD is highly prevalent. Therefore, systematic attention to this issue is essential in treatment.

Existing literature supports an association linking IR to depressive disorders. Fernandes et al. have shown that IR, a critical pathophysiological feature of diabetes, also serves as a metabolic substrate for depression-related disorders and depression itself (25). According to Watson et al., IR is a characteristic feature of the depressive state, a finding that solidifies the link between IR and persistent major depression (15). However, research conducted by Junjun Liu et al. provides evidence for a nonlinear relationship between the TyG index, a surrogate marker for insulin resistance, and psychotic symptoms in adults with MDD, suggesting a potential metabolic link that is specific to depression with psychotic symptoms (16). A key advantage of the TyG index for IR assessment is its basis in accessible routine measurements—fasting triglycerides and glucose—and the ease of computation. Relevant studies have found that the TyG index shows superior accuracy and reliability in assessing IR compared to other widely used methods, such as the Homeostasis Model Assessment of Insulin Resistance (HOMA-IR), which is based on fasting glucose and insulin levels (26). In the studies conducted by Son et al., the TyG index demonstrated superior performance in identifying IR (27).

A potential mediating pathway for the TyG index-depression interaction in the development of psychotic symptoms is likely rooted in inflammatory processes. Relevant studies have demonstrated that various inflammatory cytokines are strongly associated with the onset of depression, and individuals with elevated early-life inflammatory markers are at a higher risk of developing psychotic symptoms (28–30). Chang et al. found that there is a correlation between lipids, inflammatory markers, and IR (31). Our investigation revealed a robust association between elevated TyG index levels and increased risk of psychotic symptoms. In multivariate analysis (Model 3), participants in the highest TyG tertile (T3) had over twice the odds of psychotic symptoms relative to those in the lowest (T1) (OR = 2.138, 95% CI: 1.526, 2.994; **Table 3**). This finding was reinforced by a non-linear dose-response curve (**Figure 1**), demonstrating a threshold effect at TyG ≥ 8.06 in adolescents with MDD. Mirroring our results, studies in adults with MDD have reported a comparable threshold, corroborating a TyG value around 8.42 for symptom risk (16). The differences may be attributed to factors such as the age of the study population and whether the patients are experiencing their first episode and are untreated. These findings indicate that the TyG index could be a useful clinical indicator for identifying MDD patients at risk of developing psychotic symptoms.

The precise mechanisms connecting IR and depression continue to be explored. Research indicates that insulin resistance (IR) may exert its effects by influencing the central nervous system, which includes the direct regulation of dopaminergic and serotonergic neurotransmission, as well as inducing sustained dysregulation of the HPA axis; both mechanisms are critical components of the pathophysiological processes involved (32, 33). The dysregulation of the HPA axis initiates a metabolic cascade: it contributes to lipid abnormalities and visceral adiposity, while cortisol excess can promote hyperlipidemia through increased lipolysis and lipoprotein synthesis (34). These lipid alterations, marked by high triglycerides, drive the release of free fatty acids, which in turn induces IR in non-adipose tissues (35). HPA axis dysregulation also directly compromises insulin sensitivity, exacerbating metabolic dysfunction (36). The TyG index, by quantifying triglycerides and fasting glucose, effectively captures key components of this entire process.

Given the clinical relevance of IR to psychotic symptoms and depression, targeting IR may offer a potential therapeutic strategy. For instance, studies have shown that pioglitazone can stimulate peroxisome proliferator-activated receptor-γ (PPAR-γ) to reduce IR in patients with depression (37). IR can be managed not only through pharmacological interventions but also through lifestyle changes. The study by Martins and colleagues demonstrated that the Mediterranean diet effectively reduces IR (38). Similarly, Tettamanzi and colleagues provided evidence that a high-protein diet also has a beneficial effect on IR (39). Additionally, aerobic exercises of prolonged duration, such as brisk walking and swimming, and anaerobic exercises of shorter duration, such as sprints and weightlifting, can stabilize blood glucose levels and reduce IR through the interaction of exercising muscles with distant organs and tissues (such as adipose tissue, liver, cardiovascular system, and brain) (40, 41). Together, these findings suggest that a multi-faceted approach, incorporating both pharmacological and non-pharmacological interventions, may offer a more effective strategy for managing IR in patients with depression.

A principal strength of this research lies in its substantial sample size and rigorous analytical approach, employing multivariable logistic regression and RCS, which together yield substantial evidence for the TyG index-psychotic symptoms relationship in adolescent MDD. However, several limitations must be noted. Firstly, the observational design inherently limits causal interpretation of the associations. Secondly, the use of a single-center cohort compromises the generalizability of our findings, including the critical threshold. Thirdly, patients using psychotropic medications were not excluded. Fourthly, due to the lack of systematic collection of physical activity related data, this study was unable to assess and adjust for the potential impact of this factor on the results. Moreover, the possibility of residual confounding, despite extensive adjustments, precludes definitive conclusions. Finally, the sample of this study comprised a higher proportion of females, and caution should be exercised when generalizing the results to male populations. Therefore, well-designed multicenter randomized controlled trials incorporating additional covariates are crucial to validate the relationship between psychotic symptoms and the TyG index in adolescents with MDD.

In conclusion, the current study identified a J-shaped association between the TyG index and psychotic symptoms in adolescents with MDD, with an estimated threshold value of approximately 8.06. The typical J-shaped relationship suggests the presence of a critical threshold between the TyG index and psychotic symptoms: a significant positive correlation emerges once the TyG index exceeds this inflection point, whereas no significant association is observed below it. These findings provide a valuable preliminary report. however, further research is needed to establish a causal relationship between psychotic symptoms and the TyG index.

## Data Availability

The raw data supporting the conclusions of this article will be made available by the authors, without undue reservation.
